# Targeting Mycobacterial F-ATP Synthase C-Terminal α Subunit Interaction Motif on Rotary Subunit γ

**DOI:** 10.3390/antibiotics10121456

**Published:** 2021-11-26

**Authors:** Amaravadhi Harikishore, Chui-Fann Wong, Priya Ragunathan, Dennis Litty, Volker Müller, Gerhard Grüber

**Affiliations:** 1 School of Biological Sciences, Nanyang Technological University, 60 Nanyang Drive, Singapore 637551, Singapore; amaravadhi@ntu.edu.sg (A.H.); CHUIFANN001@e.ntu.edu.sg (C.-F.W.); rpriya@ntu.edu.sg (P.R.); 2 Molecular Microbiology and Bioenergetics, Institute of Molecular Biosciences, Johann Wolfgang Goethe University Frankfurt/Main, Max-von-Laue-Str. 9, 60438 Frankfurt, Germany; Dlitty@em.uni-frankfurt.de (D.L.); vmueller@bio.uni-frankfurt.de (V.M.)

**Keywords:** tuberculosis, mycobacteria, F-ATP synthase, bioenergetics, inhibitor, pharmacophore

## Abstract

Mycobacteria regulate their energy (ATP) levels to sustain their survival even in stringent living conditions. Recent studies have shown that mycobacteria not only slow down their respiratory rate but also block ATP hydrolysis of the F-ATP synthase (α_3_:β_3_:γ:δ:ε:*a*:*b*:*b’*:*c_9_*) to maintain ATP homeostasis in situations not amenable for growth. The mycobacteria-specific α C-terminus (α533-545) has unraveled to be the major regulative of latent ATP hydrolysis. Its deletion stimulates ATPase activity while reducing ATP synthesis. In one of the six rotational states of F-ATP synthase, α533-545 has been visualized to dock deep into subunit γ, thereby blocking rotation of γ within the engine. The functional role(s) of this C-terminus in the other rotational states are not clarified yet and are being still pursued in structural studies. Based on the interaction pattern of the docked α533-545 region with subunit γ, we attempted to study the druggability of the α533-545 motif. In this direction, our computational work has led to the development of an eight-featured α533-545 peptide pharmacophore, followed by database screening, molecular docking, and pose selection, resulting in eleven hit molecules. ATP synthesis inhibition assays using recombinant ATP synthase as well as mycobacterial inverted membrane vesicles show that one of the hits, AlMF1, inhibited the mycobacterial F-ATP synthase in a micromolar range. The successful targeting of the α533-545-γ interaction motif demonstrates the potential to develop inhibitors targeting the α site to interrupt rotary coupling with ATP synthesis.

## 1. Introduction

Tuberculosis (TB), an infectious disease caused by *Mycobacterium tuberculosis* (*Mtb*), accounts for more than 1.7 million deaths [1]. Over the years, the emergence of multi-drug resistant (MDR) TB (82%) towards the first line of therapy, particularly, towards Rifampicin (RR), and the incidence of latent TB infection (23% of the world’s population) present a perplexing global challenge to combat [1]. Bedaquiline (BDQ), which inhibits oxidative phosphorylation in the pathogen, is a recent addition to TB treatment against MDR [2,3]. Although BDQ has pharmacological and toxicological liabilities [4,5] and is shown to inhibit human mitochondrial F-ATP synthase [6], it demonstrated that the mycobacterial F-ATP synthase is a potent anti-TB drug target. This is also confirmed by recent discoveries of the novel mycobacterial F-ATP synthase inhibitors GaMF1, which is bactericidal and targets the mycobacterial extra loop of the rotary γ subunit [7] or EpNMF1/epigallocatechin gallate (EGCG), which inhibits the mycobacterial engine by binding to subunit ε and preventing coupling [8,9].

**Figure 1:**
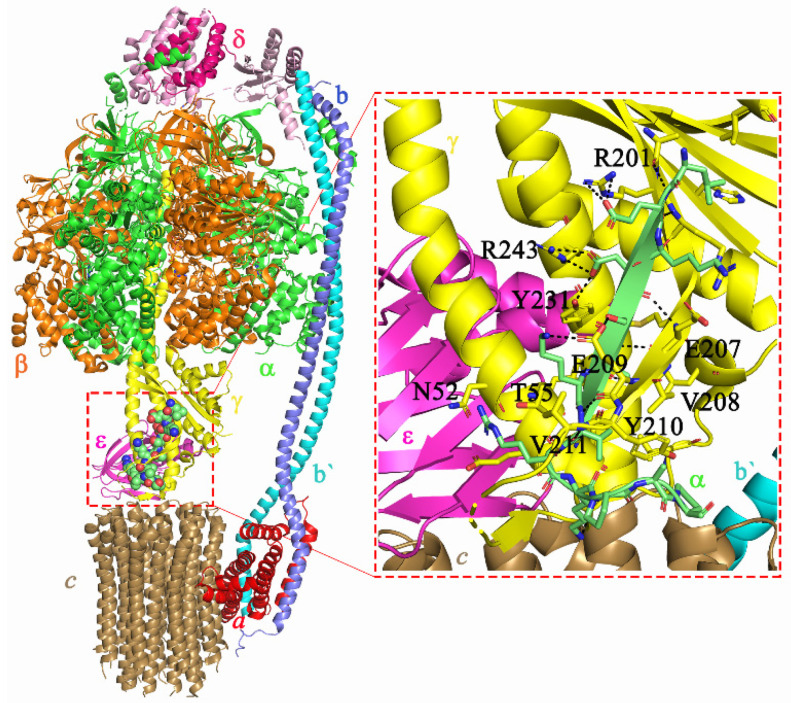
Structural model of the *M. smegmatis* F-ATP synthase with peptide α533-545 locked into the γ subunit. Shown in black lines are strong polar interactions and main chain interactions of α533-545 with γ residues.

The mycobacterial F-ATP synthase (F_1_F_O_ ATP synthase; Figure 1) contains the F_1_ subunits α_3_:β_3_:γ:ε, the H^+^-translocating F_O_ domain subunits *a*:*c_9_*, and the peripheral stalk subunits *b*:*b’*:*δ*, which holds both domains together [10,11]. Proton conduction via the subunits *a*-*c* interface and ATP formation within the α_3_:β_3_ hexamer are coupled by the rotary central stalk subunits γε [11]. A special feature of the mycobacterial F-ATP synthase is its inability to establish a significant H^+^-gradient during ATP hydrolysis, and its latent ATPase activity [12,13], which is mainly regulated by the mycobacterial extra C-terminus of the nucleotide-binding subunit α [11,14,15]. Chromosomal deletion mutation of the α C-terminal mutant ∆α514-548 stimulated ATP hydrolysis of inverted membrane vesicles (IMVs) and reduced ATP synthesis [14]. Similarly, deletion of mutants ∆α523-549 and ∆α538-549 of the *M. smegmatis* recombinant F_1_-ATPase have deciphered the main epitopes of subunit α’s C-terminus causing latent ATPase activity [15], which was further confirmed and visualized by the recent cryo-EM structure, showing that the α533-545 was trapped inside the γ subunit, forming a lock to stall the rotation of rotary elements in the *M. smegmatis* F-ATP synthase [11].

These mechanistic and structural details have provided a platform to generate a receptor-peptide-based pharmacophore on the unique interactions of mycobacterial α’s C-terminus and γ with the aim, to discover a mycobacterial ATP synthesis inhibitor. Database screening to map at least six features in the peptide-based pharmacophore provided a focused library. Subsequently, absorption, distribution, metabolism, excretion, and toxicity (ADMET) property calculations, molecular docking with both standard precision (SP), and extra precision (XP) scoring methods were performed to identify potential binders. Based on XP docking scores and molecular interaction patterns, which match α533-545 to subunit γ, eleven hit molecules were selected for experimental studies. Our ATP synthesis assays on *M. smegmatis* IMVs and recombinant F-ATP synthase reconstituted into proteo-liposomes led to the discovery of the novel mycobacterial F-ATP synthase inhibitor, AlMF1, which potently inhibited ATP synthesis with a 72% inhibition at 50 µM in recombinant *Ms*F-ATP synthase mediated ATP synthesis.

## 2. Results and Discussion

### 2.1. Homology Modeling

The recent cryo-EM structure of the *M. smegmatis* F-ATP synthase (PDB: 7JG5) [11], which has 78.69% identity at sequence level to *Mtb* enzyme, was used as a template to generate the *Mtb* subunit γ (*Mtb*γ) model (Supplementary Figure S1). Despite the lack of coordinates for the loop elements of the *M. smegmatis* subunit γ cryo-EM structure (PDB: 7JG5) [11] such as A165-L163 (13 aa) and V214-L221 (8 aa), an 81.36 % structural identity to the *Mtb* γ-sequence was computed. This enabled the generation of a good quality homology model using prime tools in the Schrödinger suite of programs. Protein quality evaluation with a procheck/Ramachandran plot showed that most of the residues were in the most favored and allowed regions with only four loop residues (G168, D170, G176, I215) being in the disallowed region (Supplementary Figure S2). The subunit α-binding motif on subunit γ was not in the vicinity of these loop residues and henceforth, the *Mtb* γ-model was used for simulation studies.

### 2.2. Receptor-Ligand-Based Pharmacophore Model

The *M. smegmatis* α533-545 peptide binds to the rotary γ subunit by both hydrophobic and polar interactions (Figure 1, [11]). Residue αL533 undergoes hydrophobic interactions with the γ residues γL103, γL106, γH184, and γR02. Likewise, the terminal residues of α subunit (αV540, αP543, αP545) were also engaged in hydrophobic interactions with amino acids γA58, γA59, γL63, and γY210. In addition to these hydrophobic interactions, amino acids αE534 and αE536 were involved in polar interactions with γ residues H201, Y239, and R243, respectively. Similarly, αS537 and αK539 were engaged in polar interaction with side-chain atoms of γE209. Furthermore, the main chain amide atoms of αK539 and αV540 interact via hydrogen bonding with main chain atoms of γE209 and γY210.

**Figure 2:**
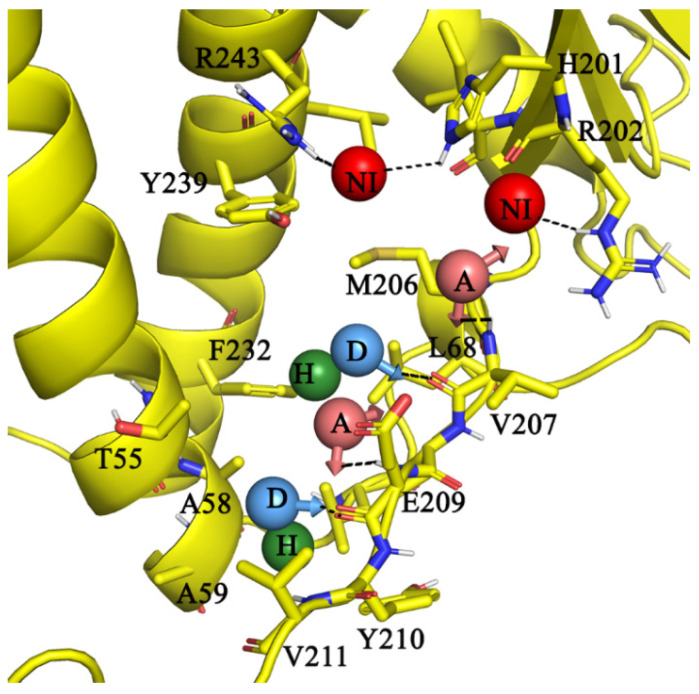
Receptor-ligand pharmacophore model. Two negative ionizable (NI) targeting amino acids γR243, γH201, and γH202; two acceptors (A) features anchoring the “NH” main chain atoms of γV207, γE209, two donor (D) features targeting the “NH” atoms of γV207 and γE209. Two hydrophobic (H) features in the vicinity of γM206, γV208, γF232, γL236, and γY210.

Based on the above-described α peptide (ligand) interaction pattern, a receptor-ligand pharmacophore was generated, which was mainly composed of eight features targeting the γ subunit residues (Figure 2). On one side of γ, two negative ionizable groups (red spheres) target amino acids γR243, γH201, and γH202, respectively (Figure 2). Two acceptors (A, light red arrowed spheres) and two donor features (D, blue arrowed sphere) anchor the “CO”/“NH” main chain amide atoms of residues γV207 and γE209, respectively. There are two hydrophobe features (green spheres)—one at the vicinity of γM206, γF232, γL236, and the other at γY210 and γV211.

#### Validation of Pharmacophore and 3D Ligand Database Screening

The ability of the eight-feature pharmacophore to discern the wild-type peptide or that of a decoy data set of MIC active molecules of the GSK TB open-source inhibitor library was carried out with phase tools [16]. Our results showed that the pharmacophore with a constraint to map at least six features was able to map the known peptide (WT) and about 300 out of 723 decoy GSK TB active molecules [17]. It is important to highlight that the decoy active library molecules are not known to bind as α-peptide to subunit γ. Nevertheless, it does provide a qualitative assessment as to whether the pharmacophore has the ability to map mycobacterial inhibitory molecules that could putatively bind to the α C-terminus motif site on γ. Such potent binding ligand(s) could abrogate the protective role conferred from the binding of the α C-terminus motif to the γ subunit. Bedaquiline (BDQ), which binds to a-c9-ring of the F_O_ sector [11], was employed as a negative test set to assess whether the pharmacophore with six feature mapping distinguishes the non-binder. Our results confirm that the pharmacophore could not map or retrieve BDQ. This assay underlines that the pharmacophore of the α-peptide motif has the ability to discern the pharmacophore features that could be vital to binding to subunit γ selectively as that of the α533-545 peptide. We next proceeded to database screening of 1.5 million molecules from the commercial Chemdiv library [18] with mapping to at least six features of the C-terminus α-peptide pharmacophore, we retrieved about 10,000 ligands. Thus, the obtained focused library was then taken into steps of virtual screening, including (i) profiling of pharmacokinetic properties such as absorption, distribution, metabolism, and toxicity (ADMET), (ii) docking, and (iii) scoring.

### 2.3. Virtual Selection of Eleven Ligands

Next, Qikprop property profiling enabled us to filter out molecules with undesirable pharmacokinetic properties. Mainly, Lipinski‘s Rule of five [19] and Veber’s rule [20] helped to enhance the drug likeliness of screening molecules. Ligands with more than two violations were filtered out. In addition, pharmacokinetic properties such as solubility (Log S > −4) snd percent of oral absorption (70–100%) indicated a good to moderate solubility and absorption through the gastrointestinal tract. Molecules, predicted to bind to human serum albumin proteins (log Khsa: −1.5 to 1.5), crossing into the blood–brain barrier with deviations from the suggested ideal log BB range of −3–1.2, being CNS active (CNS: −1/−2), or ligands, which could act as a substrate to multiple metabolic enzyme pathways (metab: >7), were excluded. Finally, a property filtered library of about 4000 ligands (Supplementary Figure S3) was taken forward to standard precision (SP) and extra precision (XP) scoring [21,22] (Supplementary Figure S3). Pose analysis of ligands with a minimum dock score of -5 kcal/mol (comparable to 10 µM) was chosen as a cut-off filter to select the best pose or hit molecules. Taken together, pose interaction analysis, XP dock scores, and favorable ADMET traits provided eleven ligands (Supplementary Table S1) that were able to interact with complementary residues of α535-545 on subunit γ.

### 2.4. Potency and Target Specificity of Ligand AlMF1

**Figure 3:**
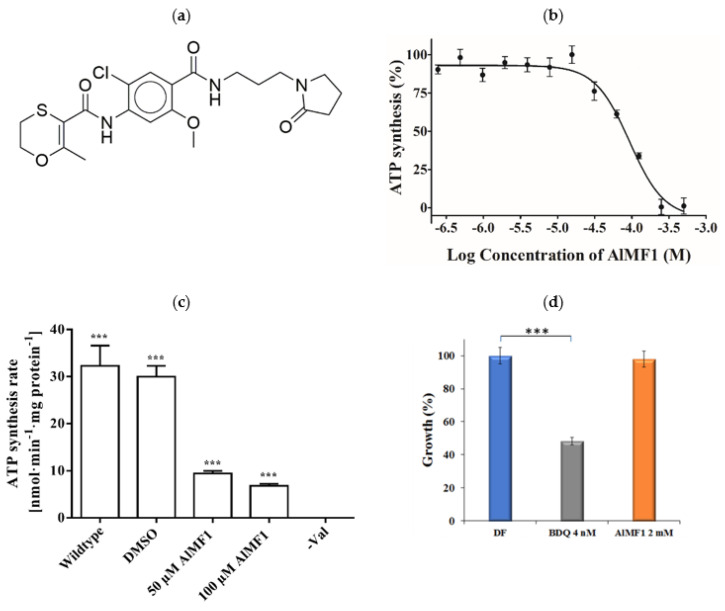
Characteristics of the mycobacterial F-ATP synthase inhibitor AlMF1. (**a**) Chemical structure of AlMF1. (**b**) Inhibition of NADH-driven ATP synthesis by AlMF1 in M. smegmatis IMVs. *** *p* < 0.0001, statistical analysis was carried out using one-sample t- and Wilcoxon test. The experiment has been carried out three times in triplicates (IC_50_ ± mean SD). (**c**) Effect of AlMF1 on ATP synthesis of reconstituted M. smegmatis F-ATP synthase. The inhibitory effect of 50 μM and 100 μM AlMF1 of M. smegmatis F-ATP synthase, which was reconstituted into proteoliposomes. (**d**) Test of possible growth inhibition of M. smegmatis by AlMF1. BDQ was used as a control. For both (**c**,**d**) the experiment has been carried out in triplicates. *** *p* < 0.0001, statistical analysis was carried out using one-way ANOVA (analysis of variance).

We evaluated the ability of the eleven Chemdiv library compounds to inhibit mycobacterial ATP synthesis using IMVs of WT *M. smegmatis* at 1 µM and 100 µM (Supplementary Figure S4). Among these compounds, N-(2-chloro-5-methoxy-4-((3-(2-oxopyrrolidin-1-yl)propyl)carbamoyl)phenyl)-2-methyl-5,6-dihydro-1,4-oxathiine-3-carboxamide (Figure 3a), called AlMF1, inhibited NADH-driven ATP synthesis of IMVs of WT *M. smegmatis* with a half-maximal inhibitory concentration (IC_50_) of 96.4 ± 3 µM (Figure 3b). When AlMF1 (50 μM) was tested against the reconstituted *Ms*F-ATP synthase, a 71% inhibition (9.2 ± 0.6 nmol·min^−1^ (mg protein)^−1^) was calculated (Figure 3c). An increase of AlMF1 to 100 µM showed increased inhibition of the enzyme of about 80% (6.5 ± 0.4 nmol·min^−1^ (mg protein)^−1^), underlining that AlMF1 targets the mycobacterial F-ATP synthase. The target specificity was confirmed by the absence of ATP synthesis inhibition of *Escherichia coli* IMVs in the presence of AlMF1 (Supplementary Figure S5a). To determine whether AlMF1 inhibits mycobacterial growth, inhibition experiments were carried out using the *M. smegmatis* mc^2^ 155 strain. As revealed in Figure 3d, the F-ATP synthase inhibitor AlMF1 did not inhibit the growth of the bacterium up to a concentration of 2 mM, while the control of BDQ showed clear inhibition at the MIC_50_ concentration used. In addition, AlMF1 did not affect the intracellular ATP level (Supplementary Figure S5b), this may indicate that the compound does not reach the required intrabacterial concentrations to exert antimicrobial activity. This is a well-known limitation of target-based approaches and has been described in the context of the pantothenate kinase inhibitors [23], including problems like limited cell envelope penetration, presence of efflux pumps, and intrabacterial metabolism [23].

### 2.5. Molecular Interactions of AlMF1 with Mycobacterial Subunit γ

**Figure 4:**
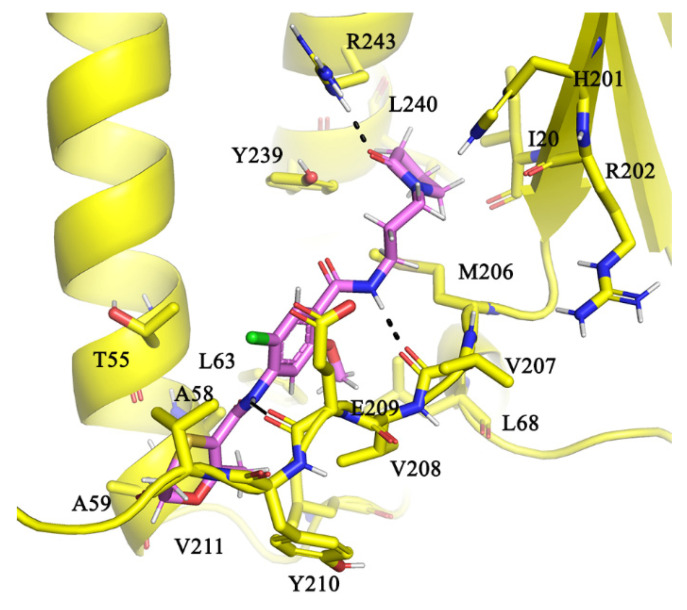
Binding pose of AlMF1. AlFF1 with its 2-oxopyrrolidin-1-yl moiety interacts with R243 (black dotted lines) while the 2-methyl-5,6-dihydro-1,4-oxathiine and the central 2-chloro-5-methoxy pheny-4-yl ring moiety were engaged in hydrophobic interactions with the subunit γ residues A59, Y210, V211, A58, L68, V207, V208, and F232. The amide atoms linking these three groups mediate hydrogen bonding interactions with carbonyl main chain atoms of γV207 and γE209.

AlMF1 with a CLogP of 1.24 has three rings 2-oxopyrrolidinyl, phenyl, and 2-methyl-5,6-dihydro-1,4-oxathiine in its structure (Figure 3a). Our molecular docking results revealed that the AlMF1 fits into a region occupied by the peptide α533-548 and interacts with a glide dock score of –5.2 kcal/mol (Figure 4). On one hand, the 2-oxo group on the pyrrolidine moiety interacts with the positively ionizable “NH” atoms of amino acids γR243 and also mediates the hydrophobic interactions with residues γY239 and γH201. The N-propyl-amino-carbonyl linker with its “NH” atoms mediates hydrogen bonding interactions with carbonyl main chain atoms of γV207. The central 2-chloro-5-methoxy pheny-4-yl ring was engaged in hydrophobic interaction with γA58, γL68, γV207, γV208, and γF232. The amide NH atoms linking the central ring to the terminal 2-methyl-5,6-dihydro-1,4-oxathiine ring further stabilize the binding with hydrogen bonding interaction with NH amide main chain atoms of γV208. Additional hydrophobic interaction of 2-methyl-5,6-dihydro-1,4-oxathiine ring with γA59, γY210, and γV211 further supports the ligand binding at this α binding site on subunit γ.

## 3. Conclusions

**Figure 5:**
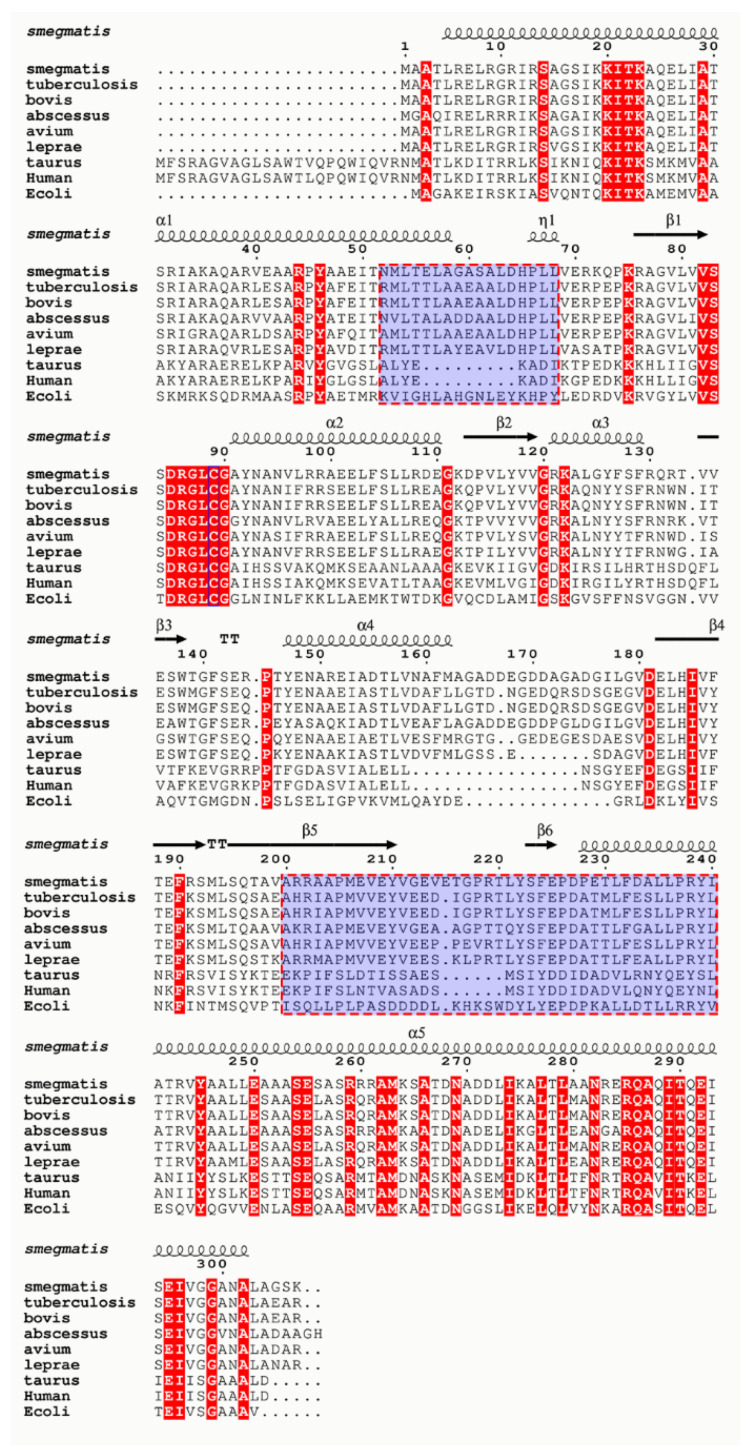
Sequence alignment of Mycobacterial γ subunits in comparison with bovine and human sequences. Highlighted in the light blue hashed boxes are the subunit α C-terminal interaction motifs on subunit γ, which are entirely unique in mycobacterial species.

Mycobacteria have adapted to survive in stringent living conditions by enhancing multiple pathways such as repressing their respiration, re-engineering surrogate enzyme expressions, or efflux pathways to rescue their survival. In this study, we have attempted to target one such pathway, which was shown to repress mycobacterial respiration or conserve ATP by engineering a subunit α C-terminus-mediated lock on subunit γ to stall ATP synthesis. A receptor-ligand pharmacophore was developed based on the interaction fingerprint of α533-548 interaction with γ. Database screening, molecular docking studies, and ATP synthesis inhibition assays have led to the identification of the inhibitor AlMF1, targeting the mycobacterial α-γ interface, with an amino acid composition not seen in the human α and γ counterparts (see Figure 5), and ensuring on-target inhibition. AlMF1 inhibited the NADH-driven ATP synthesis as well as the reconstituted mycobacterial F-ATP synthase, underlining the enzyme target specificity of the compound, which was supported by molecular docking studies. The successful targeting of the α subunit highlights the potential to advance this epitope of the molecular engine as a new area for the development of anti-mycobacterial F-ATP synthase inhibitors.

## 4. Materials and Methods

### 4.1. Modeling Simulations

*Homology modeling and protein preparation:* Homology models of *Mtb* F-ATP synthase subunits α and γ were built based on the *M. smegmatis* coordinates (7JG5; [11]). Missing loops and side-chain atoms were modeled using prime tools in the maestro Schrödinger suite of programs [24]. The resulting α-γ subunit assembly was further refined by energy minimization until the heavy atoms converged to 0.3 Å rmsd using OPLS (Optimized Potentials for Liquid Simulations) force field in protein preparation wizard module in maestro Schrödinger suite of programs [24,25].

*Receptor-ligand-based pharmacophore model:* Utilizing the coordinates of αL533-P545 C-terminal peptide interactions with subunit γ, that locked the rotary movements of γ, was used to develop a receptor-ligand pharmacophore model using Phase tools of the maestro Schrödinger suite of programs [16].

*3D ligand database preparation:* ChemDiv vendor library [18] was employed using the default settings in phase ligand preparation [24] and by checking skip reactive functional groups in ligand filtering options. The Qikprop tool [24] was used to calculate the ADMET properties of the focused library, which was obtained from the pharmacophore screen.

*Docking and scoring:* Using the α515-538 peptide, the site point was defined to generate grid, using the default setting in receptor grid generation tool in maestro suite of programs 2020. Database screening resulted in a focused library which was for molecular docking using standard precision/extra precision docking methods and corresponding scoring functions [21,22]. Ligands with the best glide dock scores were assessed for molecular interactions as detailed with peptide interaction pattern with subunit γ led to the selection of eleven hit molecules.

### 4.2. Preparation of M. smegmatis and E. coli Inverted Membrane Vesicles (IMVs)

Preparation of WT *M. smegmatis* and *E. coli* IMVs, respectively, was done as previously described by Hotra et al. (2016) [13].

### 4.3. ATP Synthesis Assay Using IMVs

ATP synthesis of the IMVs was quantified with the CellTiter-Glo^®^ Luminescent Cell Viability Assay Kit (Promega, WI, USA) as per the manufacturer’s protocol. In brief, 100 μM of compound or DMSO was spotted on individual flat-bottom 96-well microtiter plates (Corning, NY, USA). Next, 5 μg/mL of IMVs and assay buffer (50 mM MOPS, pH 7.5, 10 mM MgCl_2_, 10 μM ADP, 250 μM Pi, and 1 mM NADH) were added to the wells. The reaction was allowed to proceed for 30 min at room temperature prior to the addition of the CellTiter-Glo^®^ reagent. The plates were subsequently incubated for another 10 min in dark, at room temperature. The luminescence was measured using a Cytation^TM^ 5 (BioTek, Winooski, VT, USA) plate reader with the following parameters: luminescence; integration time, 500 ms; attenuation, none; temperature, 25 °C. Dose-response inhibition (IC_50_ ± mean SD) in triplicate was calculated by non-linear regression using log(inhibitor) vs. response with variable slope (four parameters) model in the GraphPad Prism 8 software. The statistical analysis was carried out using one-sample t- and Wilcoxon tests [26].

### 4.4. Reconstitution and ATP Synthesis of Recombinant M. smegmatis F-ATP Synthase

Recombinant *M. smegmatis* WT F-ATP synthase was purified following the protocol in Saw et al. (2020) [27]. The purified enzyme was reconstituted into small unilamellar vesicles, which were generated from Phosphatidylcholine type II S soybeans (Sigma-Aldrich, Steinheim, Germany) as described recently [28]. Proteoliposomes containing the reconstituted F-ATP synthase were collected by centrifugation (Beckman Optima L90-K, 70.2 Ti rotor, 150,000× *g*, 30 min) and the liposomes were resuspended in ATP synthesis buffer (100 mM Tris, 100 mM maleic acid, 5 mM MgCl_2_, 150 mM NaCl, 200 mM KCl, 5 mM KH_2_PO_4_, pH 7.5). ATP synthesis was measured at 37 °C by a continuous luciferase assay, monitoring the emitted light in a luminometer (FLUOstar Omega, BMG Labtech, Ortenberg, Germany). ATP synthesis measurement was carried out on white flat-bottomed 96-well microtiter plates. A total of 375 µL of the proteoliposomes, containing the reconstituted *Ms*F-ATP synthase and 20 µL ATP Bioluminescence Assay Kit CLS II (Roche Diagnostics, Rotkreuz, Switzerland), were mixed in the individual wells for 3 min at 37 °C and a baseline was recorded for 3 min at 37 °C. After preincubation, ATP synthesis was started by the addition of 2 µM valinomycin (Sigma-Aldrich) to induce a ΔΨ and 5 mM ADP (final concentration each). For inhibitor studies with AlMF1 (50 and 100 μM), proteoliposomes containing the reconstituted *Ms*F-ATP synthase were additionally preincubated for 10 min at 4 °C with the respective inhibitor, before the ATP synthesis measurements were carried out as described above.

### 4.5. Bacterial Growth in Absence and Presence of AlMF1

In brief, *M. smegmatis* mc^2^ 155 (ATCC 700084) was used in this study as the parental strain. For standard cultivation, all mycobacterial strains were grown at 37 °C in Middlebrook 7H9 broth. To observe growth inhibition in the presence of the compound, mid-log-phase pre-cultures (OD600  =  0.4–0.6) were diluted to OD600 of 0.005 and subsequently, OD600 was measured after 3 days. For the sample with the presence of the compound, 2 mM AlMF1 was added to the pre-diluted sample and incubated for 3 days before OD600 measurement. Bacterial growth percentage was calculated using the formula [(OD untreated − OD treated)/OD untreated × 100]. The statistical analysis was carried out using a one-way ANOVA test in GraphPad Prism 8 software [26].

## Data Availability

Not applicable.

## Supplementary Materials

The following are available online at https://www.mdpi.com/article/10.3390/antibiotics10121456/s1, Figure S1: Sequence alignment of Mtb γ subunit with the M. smegmatis γ. Figure S2: Ramachandran plot, showing the stereochemical quality of the M. tuberculosis γ model. Figure S3: Flow chart of steps involved in virtual screening. Figure S4: Testing the potency of eleven selected compounds in inhibiting mycobacterial ATP synthesis. Figure S5: Testing the possible effect of AlMF1 on ATP synthesis of E. coli IMVs and intracellular ATP level inside mycobacteria. Table S1: Table shows the ADMET properties of ligands used in this study.
