# Supplementary material for: Targeting Mycobacterial F-ATP Synthase C-Terminal α Subunit Interaction Motif on Rotary Subunit γ
Source: Antibiotics (Basel). 2021 Nov 26;10(12):1456. doi: 10.3390/antibiotics10121456 (PMC8698299; doi:10.3390/antibiotics10121456)
Supplement: Supplementary file 1 [file antibiotics-10-01456-s001.zip › antibiotics-1443060-supplementary.pdf]

Supplementary Materials

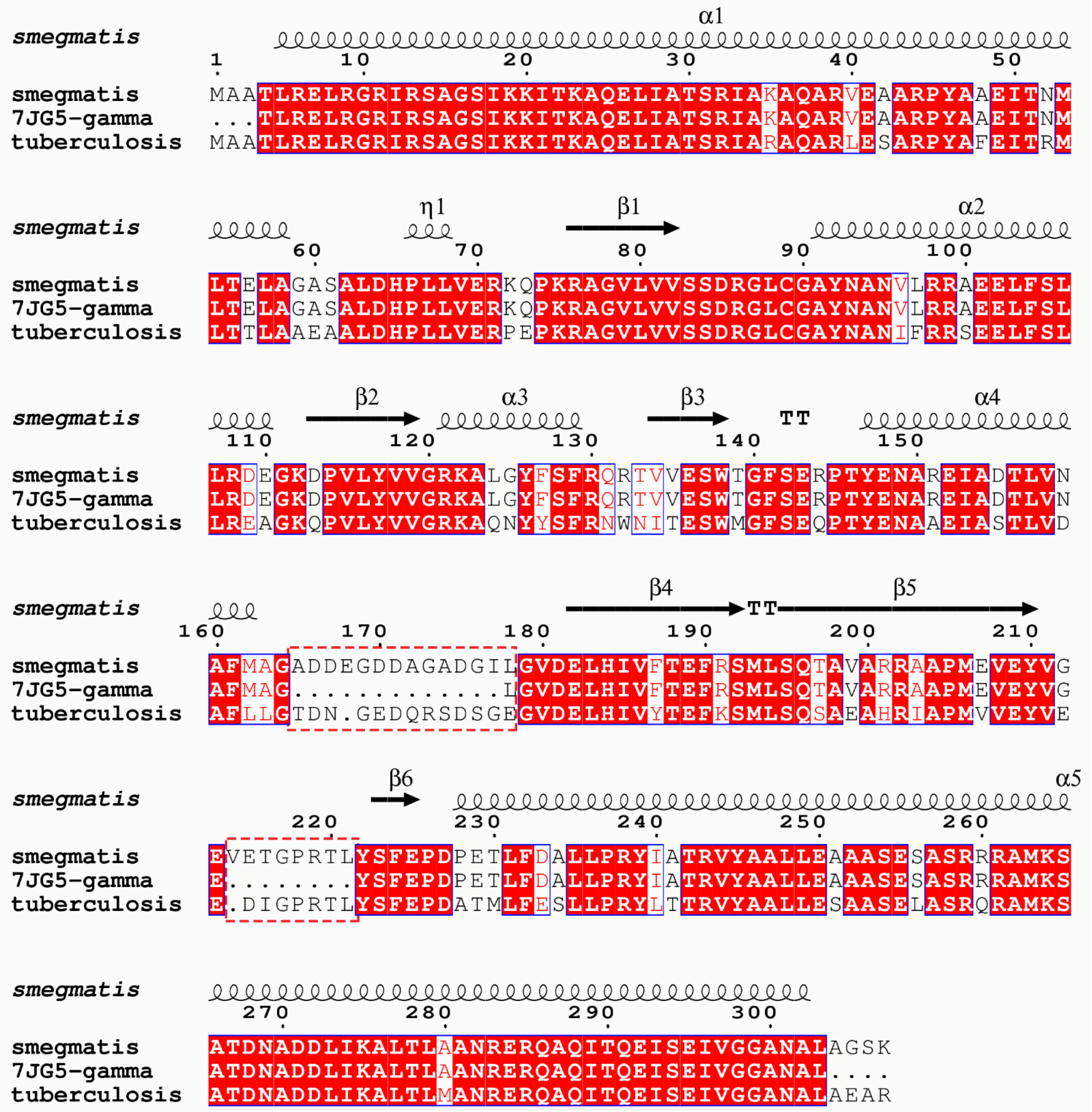

Figure S1. Sequence alignment of Mtb γ subunit with the M. smegmatis γ subunit sequence and structure sequence (7JG5:G chain). Loop elements A165-L163 (13 aa), V214-L221 (8 aa), which lacked template coordinates with the 7JG5:G chain, are shown with red colored hashed rectangle boxes.

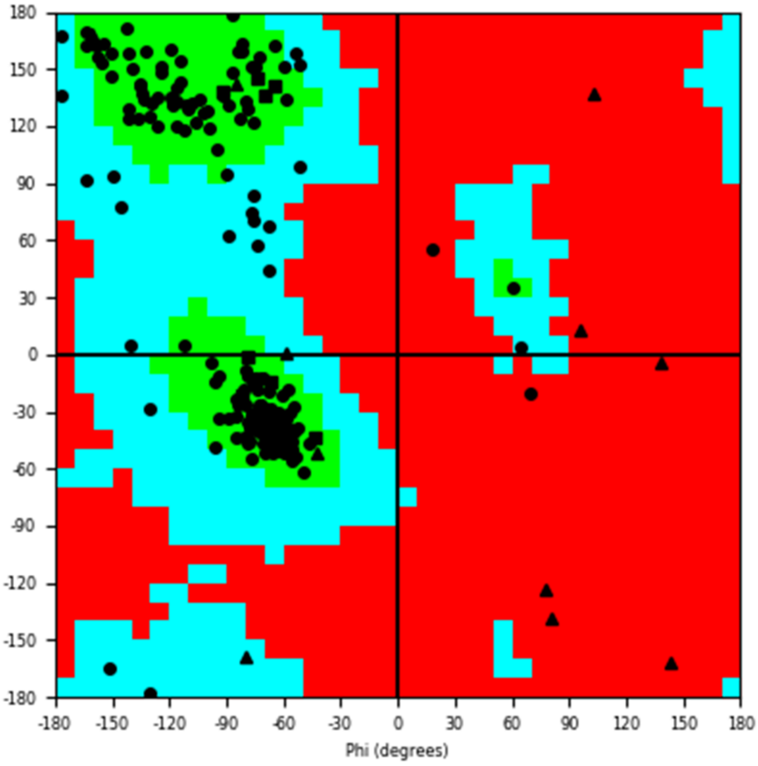

Figure S2. Ramachandran plot showing the stereochemical quality of the Mtb γ model. Most of the residues are in most favored regions (green zone), allowed (cyan) and very few residues such as G168, D170, G176, I215 from loop regions are in disallowed regions. While the other five residues appear to be also present with template structure. The data confirm that the structure, which was built from Prime homology model building tools, is reliable. (Residue markers: Triangle Glycine, Squares: Proline, Circles: All other residues).

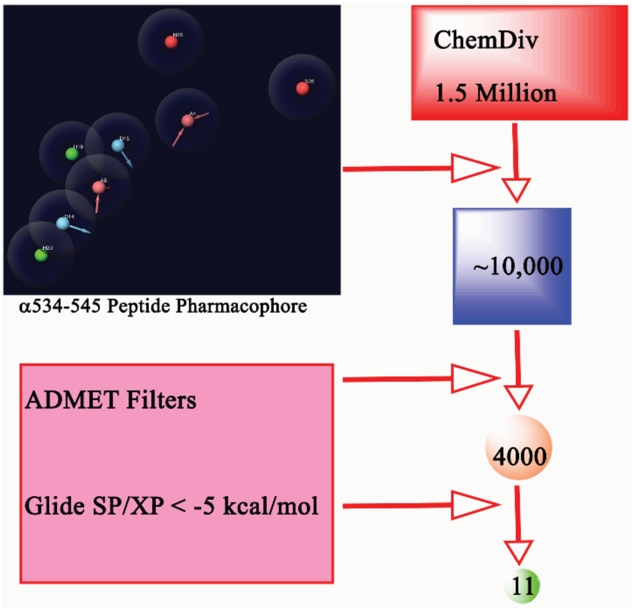

Figure S3. Flow chart showing the virtual screening steps. α533-545 peptide-based pharmacophore search on the Chemdiv database yielded about 10,000 ligands. This focused library was further enumerated with property filters, glide docking / scoring of poses with SP/ XP scoring functions. Pose selection of ligands, which match the interaction pattern of the α533-545 peptide, led to a selection of eleven molecules for experimental characterization.

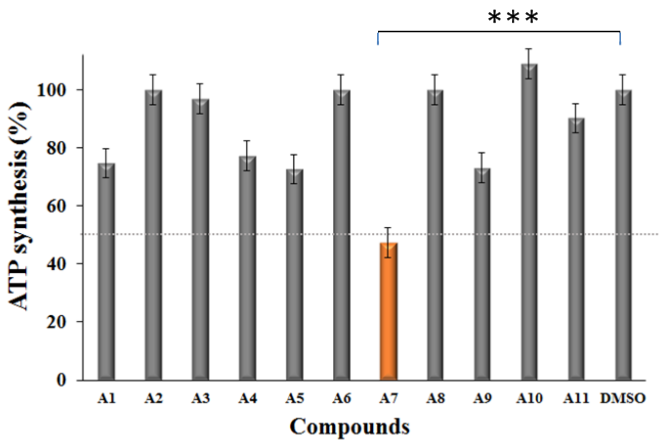

Figure S4. Testing the potency of eleven selected compounds (100 µM each) in inhibiting mycobacterial ATP synthesis of M. smegmatis IMV's. The dotted line represents the threshold of below 50% inhibition. ATP synthesis (%) in the presence of the compound is expressed as a percent of control to untreated (DMSO) [(Io-I)/Io x 100], where Io is untreated and I is treated with compound. ***: P< 0.0001, statistical analysis was carried out using one way ANOVA (analysis of variance).

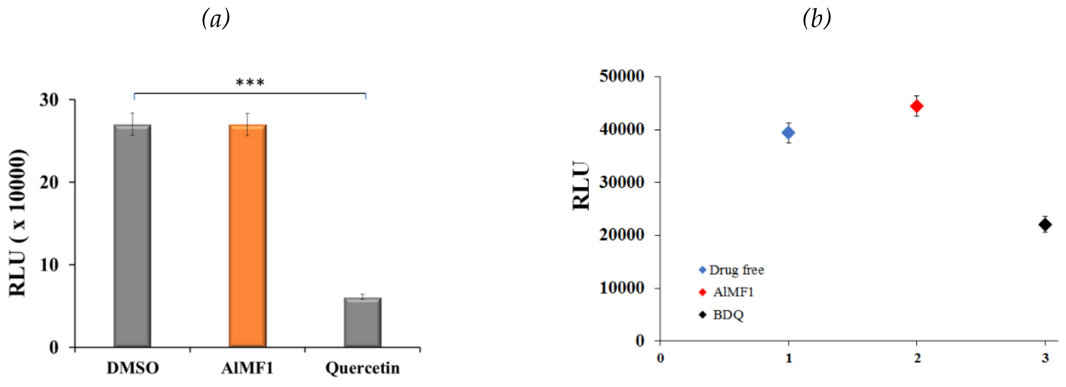

Figure S5. Testing the possible affect of AlMF1 on ATP synthesis of E. coli IMVs and intra-cellular ATP level inside mycobacteria. a ATP synthesis of E. coli IMVs in the presence of the vehicle control dimethyl sulfoxide (DMSO), 100 µM of the new compound AlMF1, and 100 µM of Quercetin as a control. The data reveal the specificity of AlMF1 as a mycobacterial F-ATP synthase inhibitor. ***: P< 0.0001, statistical analysis was carried out using one way ANOVA (analysis of variance). b Intracellular ATP level measurement of M. smegmatis in the presence of 1 mM AlMF1. No inhibitory effect of AlMF1was observed. BDQ (4 nM) as a control showed inhibitory potency. In drug free conditions, DMSO was used as vehicle control. The experiment has been carried out three-times in triplicates.

Table S1. Table shows the ADMET properties of ligands used in this study.

Sl.No	IDNUMBER	logP	logSw	XP g scores(kcal/mol)	#rotor	#rtvFG	CNS	MW	Caco	logBB	MDCK	#metab	logKhsa	%HumanOralAbsor	PSA	RuleOfFive
A1	F293-0909	3.4	-4.09	-5.5	9	0	-2	471.573	51.469	-2.017	25.477	5	0.231	81.546	121	0
A2	E918-0398	5.32	-5.7	-5.59	9	0	-2	565.671	1076.11	-1.148	535.532	7	1.465	94.301	101	2
A3	E682-0164	4.1	-5.86	-5	7	0	-2	442.55	426.706	-1.317	337.766	6	0.307	94.898	149	0
A4	C517-1579	3.38	-3.71	-5.5	10	0	-2	484.612	701.183	-1.211	490.087	5	0.224	100	87.8	0
A5	C387-0761	6.22	-2.91	-8.26	12	0	-2	499.608	1794.71	-1.147	930.868	4	1.228	100	84.3	1
A6	A0070870	5.38	-6.62	-5.54	8	0	-2	656.748	594.68	-1.327	1160.05	7	1.411	92.845	122	2
A7	E532-2866	0.99	-3.261	-5.23	8	0	-1	467.966	501.645	-0.883	1447.26	4	-0.29	90.944	111	0
A8	C387-0037	3.94	0.6886	-6.7	10	0	-1	423.511	2560.54	-0.731	1366.81	3	0.43	100	76	0
A9	C241-2052	2.69	-3.695	-6.53	10	0	-2	442.532	37.333	-2.156	84.73	6	-0.49	65.424	142	0
A10	C066-2093	1.22	0.0497	-5.3	9	0	-1	407.468	593.997	-0.968	659.237	4	-0.447	92.178	99.6	0
A11	6623-2029	3.21	-1.952	-7.23	13	1	-2	463.573	298.028	-1.874	286.512	4	0.322	100	135	0

LogP : Partition Coefficient
logSw: Water solubility
#rotor: rotatable bonds
#rtvFG: reactive functional groups
CNS: Central Nervous System
Mol_MW: Molecular Weight
logHERG: human ether-a-go-go-related gene
CaCo: Caco-2 Permeability
logBB: Blood Brain barrier
MDCK: Madin-Darby Canine Kidney-P-glycoprotein assay for Permeability/ Drug Efflux
#metab: No. of Metabolism routes
HSB: human serum binding
% human oral absorption
PSA: Polar Surface Area
